# Dietary Fibres Differentially Impact on the Production of Phenolic Acids from Rutin in an In Vitro Fermentation Model of the Human Gut Microbiota

**DOI:** 10.3390/nu12061577

**Published:** 2020-05-28

**Authors:** Jaroslav Havlik, Vittoria Marinello, Andrew Gardyne, Min Hou, William Mullen, Douglas J. Morrison, Thomas Preston, Emilie Combet, Christine A. Edwards

**Affiliations:** 1 Human Nutrition, School of Medicine, Dentistry and Nursing, College of Medical, Veterinary and Life Sciences, University of Glasgow, Glasgow G31 2ER, UK; havlik@af.czu.cz (J.H.); vittoria.marinello@lanarkshire.scot.nhs.uk (V.M.); andrew.gardyne@outlook.com (A.G.); minhou@sjtu.edu.cn (M.H.); Emilie.CombetAspray@glasgow.ac.uk (E.C.); 2 Institute of Cardiovascular and Medical Sciences, College of Medical, Veterinary and Life Sciences, University of Glasgow, Glasgow G12 8QQ, UK; William.Mullen@glasgow.ac.uk; 3 Scottish Universities Environmental Research Centre, East Kilbride G75 0QF, UK; Douglas.Morrison@glasgow.ac.uk (D.J.M.); Tom.Preston@glasgow.ac.uk (T.P.)

**Keywords:** colonic metabolism, flavonols, gas chromatography mass spectrometry, gut microbiome, phenolic acids, dietary fibre

## Abstract

Polyphenols are often ingested alongside dietary fibres. They are both catabolised by, and may influence, the intestinal microbiota; yet, interactions between them and the impact on their resultant microbial products are poorly understood. Dietary fibres (inulin, pectin, psyllium, pyrodextrin, wheat bran, cellulose—three doses) were fermented in vitro with human faeces (*n* = 10) with and without rutin (20 µg/mL), a common dietary flavonol glycoside. Twenty-eight phenolic metabolites and short chain fatty acids (SCFA) were measured over 24 h. Several phenolic metabolites were produced during fibre fermentation, without rutin. With rutin, 3,4-dihydroxyphenylacetic acid (3,4diOHPAA), 3-hydroxyphenylacetic acid (3OHPAA), 3-(3 hydroxyphenyl)propionic acid (3OHPPA) and 3-(3,4-dihydroxyphenyl)propionic acid (3,4diOHPPA; DOPAC) were produced, with 3,4diOHPAA the most abundant, confirmed by fermentation of ^13^C labelled quercetin. The addition of inulin, wheat bran or pyrodextrin increased 3,4diOHPAA 2 2.5-fold over 24 h (*p* < 0.05). Rutin affected SCFA production, but this depended on fibre, fibre concentration and timepoint. With inulin, rutin increased pH at 6 h from 4.9 to 5.6 (*p* = 0.01) but increased propionic, butyric and isovaleric acid (1.9, 1.6 and 5-fold, *p* < 0.05 at 24 h). Interactions between fibre and phenolics modify production of phenolic acids and SCFA and may be key in enhancing health benefits.

## 1. Introduction

Intake of plant phenolics is estimated to be ~1 g per day [1,2,3] in most populations, a small proportion of which are flavonols [3,4]. Daily flavonol intake varies between populations and dietary intake patterns: from ~5.4 mg per day in Finland [5], 19 mg per day Spain [6] and 34 mg per day in France [1], with 59%–79% of daily flavonol intake represented by quercetin glycosides [1,6]. Rutin is an abundant glycosidic form of quercetin, found in significant quantities in capers, black olives and buckwheat (332, 45 and 36 mg/100 g fresh food weight, respectively) [7]. Drupe fruits such as blackcurrants and raspberries may also provide up to 11 mg of rutin per 100 g [7,8]. Research on flavonols as plant food bioactives with proposed health benefits has expanded in the last two decades, with major focus on cancers and cardiovascular diseases [9,10,11,12].

The metabolism of rutin has been extensively studied ex vivo [13,14,15], in rodents [16,17,18] and in human intervention studies: after ingestion, only 14%–17% of rutin is absorbed in the small intestine [19,20]. Most ingested rutin therefore enters the colon, where it is catabolised by the gut microbiota via a pathway starting with the hydrolytic cleavage of the disaccharide moiety [19,20,21]. This, combined with fission of the C-ring of the aglycone, results in the formation of a series of phenolic acids and phloroglucinol derivatives from either of the two remaining rings [15]. There is a high degree of inter-individual variability in the metabolites produced [15,21], with age [21] and ethnicity [22], two factors shown to modify the metabolite fingerprint excreted in urine. Although 3,4diOHPAA is usually the dominant early metabolite, some individuals appear to shift metabolism towards different phenolic products [15]. Phenolic acids are absorbed from the colon and released into the circulation alongside other metabolites and are therefore speculated to be responsible for some of the biological effects of rutin [15,23]. The main metabolite, 3,4diOHPAA, has been shown to possess high biological activities in vitro with some advantages over its parent compound, such as low toxicity and solubility [24], antiplatelet aggregation properties [25], suppression of lipopolysaccharide induced pro-inflammatory cytokines TNF-α, IL-1β and IL-6 in peripheral blood mononuclear cells [26] and inhibition of protein glycation [23,27].

Much of the evidence for gut-microbiota-mediated metabolism of polyphenols to date has been assessed using in vitro batch cultures with human stool samples. The studies differ in media used. Some cultures did not contain any fermentable carbohydrates [13,15,28,29], while others did [14,30]. The presence of fermentable carbohydrates in media, however, might be an important factor changing the pattern of polyphenol catabolism, yet the results are contrasting. More rapid fermentation of rutin was seen in the presence of glucose [15] resulting in greater production of metabolites, while less total phenolic acids and 3,4diOHPAA were seen in the presence of complex carbohydrates in the medium [31]. There are several potential mechanisms by which additional fermentable carbohydrate impacts upon bacterial polyphenol catabolism. Firstly, fermentation of carbohydrates to short chain fatty acids (SCFA) results in a decrease in colonic pH [32] promoting changes in bacterial enzyme activity [33]. Secondly, some fibres may be selective in promoting growth of specific bacterial groups within the intestinal microbiota [34], thus altering polyphenol metabolism. Thirdly, dietary fibres could decrease or delay colonic polyphenol availability by altering small intestinal absorption and transit time [35]. Finally, dietary fibre may be a rich source of non-extractable polyphenols and release associated metabolites when they are fermented by the bacteria [36], which trigger antimicrobial action or inhibit bacterial hydrolases.

The evidence suggests that the polyphenol metabolites produced by the gut microbiota change (in terms of quality and quantity) in the presence of fibre. In this study, we undertook a systematic investigation of the impact of dietary fibres (pectin, inulin, psyllium, pyrodextrin, wheat bran and cellulose) on the catabolism of rutin in human faecal batch cultures, to inform food product formulation (e.g., soups, juices), towards improved products with higher health impact.

## 2. Materials and Methods

### 2.1. Chemicals

Oxygen free nitrogen (OFN) was from BOC-Linde (UK). Chemicals used as standards (Supplementary materials, Table S1), 3-(3-hydroxyphenyl)propionic acid [3,3OHPPA], 3-3,4-dihydroxyphenyl)propionic acid [3,3,4diOHPPA], 4-hydroxy 3-methoxyphenylacetic acid [4OH3MPAA] and 4-hydroxymandelic acid [4OH-mandelic acid] were from Alfa Aesar (Thermo Fisher Scientific, MA, USA), 2,4,5-trimethoxycinnamic acid [TMCA], 3-(3-hydroxy 4-methoxyphenyl)propionic acid [3,3OH4MPPA], 3-(4-hydroxy 3-methoxyphenyl)propionic acid [3,4OH3MPPA], 3-(4-hydroxyphenyl)propionic acid [3,4OHPPA], 3,4-dihydroxybenzoic acid [3,4OHBA], 3,4-dihydroxyphenylacetic acid [3,4DiOHPAA], 3-hydroxy-4-methoxycinnamic acid [isoferulic acid], 3-hydroxy-4-methoxyphenylacetic acid [3OH4MPAA], 3-hydroxybenzoic acid [3OHBA], 3-hydroxyphenylacetic acid [3OHPAA], phenylacetic acid [PAA], 3-phenyllactic [3PLA], 3-phenylpropionic acid [3PPA], 4-hydroxy 3-methoxybenzoic acid [VAN], 4-hydroxybenzoic acid [4OHBA], 4-hydroxyphenyl acetic acid [4OHPAA], benzoic acid [BA], caffeic acid, ferulic acid, mandelic acid, *p*-coumaric acid, phloroglucinol, pyrocatechol, quercetin, resorcinol and trans-cinnamic acid were purchased from Sigma Aldrich. The uniformly labelled ^13^C quercetin (>98.5% C atom labelled) was obtained from Isolife (NL). The fibres used in the study were as follows: inulin HP ([INU]; Beneo-Orafti, BE), pectin [PEC] from citrus peel and α-cellulose ([CEL]; Sigma Aldrich), wheat bran Organic ([WB]; Infinity Foods, UK), powdered psyllium husks ([ISP]; Myprotein, UK), pyrodextrin or else resistant maltodextrin [RM] was Fibersol-2 (Matsutani Chemical Industry, JP). Other solvents, buffer constituents, and reagents were from Sigma-Aldrich and were of highest available purity.

### 2.2. In Vitro Fermentation Model

#### 2.2.1. Donors, Sampling and Faecal Slurry Preparation

Faecal samples were obtained from healthy donors aged 20 to 41 years (mean age 27), with a mean BMI 26.3 kg/m^2^ (ranging 22.8–35.1). Donors were mostly male (72%), all Caucasian, had not undergone antibiotic treatment in the previous 6 months, had no history of gastrointestinal disease, and were not vegetarian or vegan. All subjects followed a low polyphenol-low fibre diet (for details, see Supplementary Materials, Figure S3) for 48 h before providing a faecal sample. The study was approved by the College of Medical, Veterinary and Life Sciences Ethics Committee, University of Glasgow (no. 2011023) and donors gave written informed consent.

Stool samples were collected using in-house collection kits consisting of a sealed 0.7 L plastic container sealed within a plastic bag with the addition AnaeroGen™ 3.5 L Sachet (Oxoid, UK) after sample collection. The samples were processed in the laboratory within 2 h of sampling. A faecal slurry (25% wt/wt) was prepared by mixing 50 g of faeces with 150 mL of sterile OFN-purged phosphate buffer, pH 7.0 using a blender and the particulate material was removed by straining through nylon mesh.

#### 2.2.2. Fibres

Fibres were pre-weighed (0.8, 1.7 and 3.3 g) into 100 mL gas-tight, crimp-top sealed fermentation bottles, to represent assay concentrations corresponding to a physiologically representative bolus intake of 5, 10 and 20 g of fibre potentially diluted in 300 mL of colonic contents [37]. INU, WB, RM and CEL were tested at all three concentrations. Only fermentations containing 0.8 and 1.7 g fibre were used for PEC and just 0.8 g for ISP due to the high viscosity. Vials containing no fibre were also included (no fibre blanks). The fermentations were carried out with and without rutin to account for any phenolics intrinsically present in the fibres.

#### 2.2.3. Faecal Incubations

Each vial contained 42 mL of fermentation medium at pH 7.0, 2 mL of reducing solution and 1 mL of rutin aqueous solution (1 mg/mL) or water for blanks [38]. In some cases, the size of the fermentation model was scaled-down by half due to low donor faecal sample weight. The rutin concentration used in the fermentations in this study was selected based on 86% rutin recovery in ileostomy subjects [19] assuming a postprandial ileal output of 162 mL within 2 h after consuming a 300 mL meal [39] and additional estimated two-fold dilution in the proximal colon by colonic secretions. Under these conditions, a moderate intake of fruit, e.g., 200 g of blackberries containing 3.89 mg/100 g [8] may result in approximately 20 µg/mL of rutin in the proximal colon.

After full decolouration of the medium, we inoculated with 5 mL of faecal slurry (total volume 50 mL) and incubated in a shaking bath at 37 °C, 60 strokes/min and sampled at 0 h, 6 h and 24 h. Aliquots (5 × 1 mL) were collected with a syringe and needle through the self-sealing septa at each timepoint and stored at −80 °C. One aliquot was used for pH determination with a calibrated pH meter (Hannah pH20 instruments, USA). Aliquots for SCFA analysis were alkalized to pH > 9 prior to storage [38,40].

#### 2.2.4. ^13^C Labelled Quercetin Study

^13^C quercetin (10 µg/mL) was fermented in parallel with native quercetin (^12^C) and corresponding controls. No fibre was added. The experiment was carried out as two independent replicates with the stool of a single donor donated within 1 month. The conditions were the same as above apart from being scaled-down to 1.5 mL of the fermentation fluid in each of the 5 mL vials. Quercetin (5 mg/mL, 3 µL in 100% methanol, 10 µg/mL in the assay) was injected into the incubation vials with a glass syringe. At each timepoint (0 h, 3 h, 6 h and 24 h), vials were taken and frozen at −80 °C. Blank cultures not containing quercetin and samples containing quercetin but not stool inoculum (labelled as INI for timepoint 0 h) were prepared in parallel. They were thawed just before extraction and spun at 6000× *g* to remove particulate material from the supernatant.

### 2.3. Fibre-Phenolic Sequestration Experiment

To investigate the effect of fibres on recovery of phenolics from fermentation samples, fibres were pre-weighed in 5 mL tubes in amounts of 8, 17 and 33 µg and subsequently hydrated with 500 µL of distilled water (pH 5.5), so that the final concentration corresponded to equivalent of 0.8, 1.7 and 3.3 g/50 mL After spiking with mixed standards, (5 µg each), solutions were incubated for 6 h at 37 °C and extracted using the same procedure as for fermentation samples.

### 2.4. Phenolic Acids Extraction

A gas chromatography-mass spectrometry (GC/MS) analysis of phenolic compounds in fermentation fluid was performed as previously described [41] with modifications. Fermentation aliquots were thawed and volumes of 500 µL transferred to 5 mL glass tubes. Internal standard solution (ISTD; 30 µL of 0.2 mg/mL 2,4,5-trimethoxycinnamic acid) was added, the mixture vortexed, acidified with 60 µL of 1M HCl, vortexed again, and left at 4 °C for 10 min. The mixture was then extracted twice with 1.5 mL of ethyl acetate. After each extraction step, the upper organic layer was removed after centrifugation at 1600 × *g* and transferred to 1.5 mL amber vials. Samples were evaporated at 45 °C until dryness (approx. 40 min) on Savant SpeedVac SPD131DDA concentrator (Thermo Fisher Scientific, MA, USA), with repetitive extractions being pooled in the same corresponding vial. After the second evaporation, vial walls were rinsed with 200 µL of dichloromethane and evaporated again for 10 min. Dry residues were derivatised under OFN, using 50 µL of N,O-Bis(trimethylsilyl)trifluoroacetamide (BSTFA) with 1% chlorotrimethylsilane (TMCS) for 4 h at 65 °C.

### 2.5. Phenolic Acid Analysis

Anhydrous hexane (99%, 400 μL) was added to each vial before analysis. A set of 13 calibration solutions, containing 28 phenolic acids, hydroxybenzenes and quercetin ranging from 1 μg to 80 μg/mL, was extracted and analysed alongside all faecal samples. The analysis was performed on a Trace GC equipped with a split/splitless injector interfaced to a DSQ mass spectrometer and an AI3000 autosampler (Thermo Fisher Scientific, MA, USA) using the following conditions: inlet temperature 250 °C, split injection (1:25), 1 μL sample volume. A Zebron^TM^ ZB-5 capillary column (30 m × 0.25 mm i.d., df = 0.25 µm) was used for separation. The carrier gas flow (He) was constant at 1.2 mL/min. The oven programme started at 140 °C (held 0.5 min), rose to 160 °C at 6 °C/min to 270 °C and was increased then at 30 °C/min to a final temperature of 320 °C (held for 4 min). The transfer line was maintained at 310 °C and ion source temperature at 270 °C. Acquisition was performed in positive electron ionization mode in full scan (m/z 50–450) with an ionization energy of 70 eV, from 1.7 to 20 min. Quercetin and ^13^C-quercetin were monitored using single ion monitoring (SIM) for ion m/z 647, 662 and 595 respectively, from 21–25 min. Identification of phenolics and quercetin was achieved by comparison with the retention times and mass spectra of authentic standards. Acquisition and analyses of GC-MS data were performed on Xcalibur version 2.1 (Thermo Fisher Scientific, MA, USA). The ^13^C-quercetin and its products were quantified using calibration curves based on quantifier ions of the corresponding non-labelled compounds in MS Excel. Differences between controls and treatments were thoroughly checked for unknown metabolites using AMDIS ver. 2.1, Compare Files Postanalysis feature. A detailed GC-MS method including conditions and basic validation is shown in Supplementary Materials, Table S2.

### 2.6. Short Chain Fatty Acid Extraction and Analysis

Short chain fatty acids (SCFA) were measured by GC-FID of acidified ether extracts of the fermentation fluid as previously described [31].

### 2.7. Statistical Analysis

Data were expressed as mean values and visualised in IBM SPSS Statistics ver. 22 (International Business Machines, NY, USA). Normality was assessed using the Shapiro-Wilk test. Due to non-normal distribution in some subsets, skewness was reduced by log10 transformation. Potential differences between controls and treatments were analysed using 3-way repeated measures analysis of covariance (MANOVA), one-way ANOVA with Dunnett’s or Tukey’s post hoc test for factors fibre (INU, PEC, ISP, RM, WB, CEL), concentration (0, 0.8, 3.3 g/50 mL of fibre), rutin (including rutin, R+; excluding rutin R-) or Time (0 h, 6 h, 24 h) on the 28 phenolic metabolites used as variables as indicated in the method section.

Areas under 24 h curve (AUC_24_) were established for compounds of interest, reflecting their total production in solution. If the solution initially contains 10 µg/mL of a compound which remains unmetabolised over time, the AUC_24_ value will be 240 µg/mL/24 h; analogously, if same compound is linearly metabolised to 0 within 24 h, it produces an AUC_24_ of 120 µg/mL/24 h.

The effects of rutin on SCFA and pH were analysed using independent samples t-test (2-tailed) for each factor with a Benjamini-Hochberg multiple testing correction. No data transformation was applied. Levene’s test was used to assess equality of variances. Results were expressed as means ± S.E.M or S.D. and were considered significant at *p* < 0.05.

## 3. Results

### 3.1. GC-MS Method Optimisation and Validation

The method was adapted from [42] and internally validated. Recoveries were determined by spiking a pooled faecal sample with phenolic acid standards at concentrations of 10 μg/mL. Average recoveries were 91%, typically ranging between 81% and 106%, however, for 4OH-mandelic acid, BA, 3PPA, PAA and the polar hydroxybenzenes, such as resorcinol or phloroglucinol recoveries were low (10%–26%). Limits of detection (signal-to-noise ratio of 3:1) for four main rutin metabolites: 3OHPAA, 3,3OHPPA, 3,4diOHPAA and 3,3,4diOHPPA were 0.2, 0.15, 0.08 and 0.25 µg/mL, respectively. Basic method validation data are shown in Supplementary Materials, Table S2.

### 3.2. Phenolics and Their Metabolites Released from Fibres

**Figure 1:**
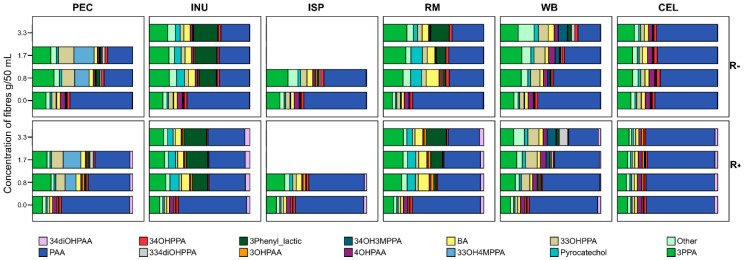
Relative profile of phenolic metabolites (rel% AUC_24_) released from fibres present in the medium in fermentation vials with rutin added (R+, 20 µg/mL) and without rutin (R-); INU, inulin, PEC, pectin, ISP, psyllium, RM, pyrodextrin, WB, wheat bran, CEL, cellulose; *n* = 10. The light pink bar refers to the main metabolite of rutin and shows its rather low proportion in the whole pool of phenolic acid metabolites present. For other abbreviations, see Materials and Methods section.

Fibres alone, even without addition of rutin released significant amounts of phenolics, which showed fibre-specific patterns (Figure 1, Table 1).

Some of these (pyrocatechol; BA; PAA; 3PPA; 3,3OHPPA; 3,3,4diOHPPA or 3,3OH4MPPA) are potential rutin catabolites. In fermentations blank or CEL (no matter if rutin was present or not), phenolic profile was similarly represented by high AUC_24_ for PAA (510–600 µg/mL/24 h) and 3PPA (118–140 µg/mL/24 h) (Figure 1, Table 1). The presence of fermentable carbohydrates except WB decreased PAA 2-fold, and increased selected phenolics in fibre-specific manner, e.g., 3PLA was highest in fermentations of INU and 3,3OHPPA and 3,3H4MPPA were more abundant when PEC was fermented. None of the fibres released 3,4diOHPAA—(the main rutin metabolite) in significant quantities except ISP which contained also very small significant amounts of quercetin (Table 1).

### 3.3. Fibre-Phenolics Sequestration Interactions

When spiking the fibre suspensions in aqueous solution (pH 5.0) with 5 µg of standards, we observed ~160% recoveries for all metabolites in the presence of PEC, as a consequence of ISTD sequestration. Recoveries of the four potential rutin metabolites (3OHPPA, 3,4diOHPAA, 3,3,4diOHPPA) except 3,3OHPAA, were significantly lower in the presence of WB than that of BLA. WB also highly reduced the recovery of non-rutin metabolites mandelic acid, caffeic acid, *p*-coumaric acid but more importantly that of quercetin and moderately that of 4OHPAA, both potential rutin metabolites (for details, see Supplementary Materials, Table S3). At the highest concentration of WB, the majority of these compounds were trapped in the matrix, resulting in poor < 4% recoveries. The most prominent effect of WB was seen on 3,4diOHPAA and quercetin, even the lowest concentration present in the sample reduced their recoveries to 8 and 13%, respectively. However, other fibres did not interfere with the recovery of the spiked standards and these were 98%–107% compared to recoveries from blank samples (Supplementary Materials, Table S3).

### 3.4. Colonic Catabolism of Rutin in a Human Batch Fermentation Model

**Figure 2:**
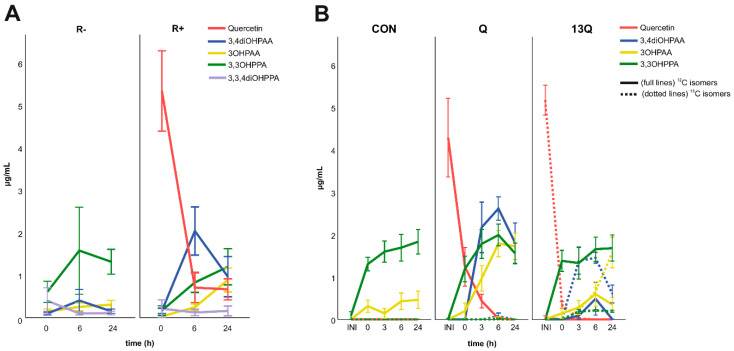
(**A**) Catabolic products of rutin in fermentations without fibres. R+, fermentation with rutin, R-, corresponding control without rutin; (**B**) Catabolic products of rutin aglycone –quercetin. CON, without inoculum and quercetin, Q, quercetin, 13Q, ^13^C all-labelled quercetin isotope. Error bars denote S.E.M., *n* = 10 (for A), n = 4 (for B). INI, initial concentration added to vials (measured in a parallel sample without faecal inoculum).

When no fibre was present in fermentations, rutin was catabolised to a limited set of products: quercetin, 3,4diOHPAA, 3OHPAA, 3,3OHPPA and traces of 3,3,4diOHPPA. The sum of the metabolites explained 52%, 23% and 21% of the original molecular mass of the quercetin aglycone at 0, 6 and 24 h, respectively. Some of the catabolites were also found in incubations without rutin. The data are shown in Figure 2A. To reveal the origin of these, the isotope-labelled experiment with ^13^C-quercetin was conducted and has shown, that approximately 40% of the phenolic acids observed in fermentations do not originate from the added compound but are formed from other substrates. While 100% of 3,4diOHPAA was carrying the label, 75% of 3OHPAA was labelled alongside 25% unlabelled at both timepoints. Of the 3,3OHPPA only 17%–25% of the metabolite found was labelled alongside the majority of unlabelled compound (Figure 2B). Only a small proportion (0.1%) of labelled 3,3,4diOHPPA was found alongside the omnipresent unlabelled 3,3,4diOHPPA (not shown in the graph). Most of the 3,3OHPPA but also a large proportion of 3OHPAA are thus formed from precursors present in medium. The mean sum of ^13^C products for all fermentation timepoints was 5.38, 3.79 and 5.57 µg/mL of the 10 µg/mL added at 3 h, 6 h and 24 h, respectively. No other ^13^C products were found.

### 3.5. Effects of Fibres on Rutin Metabolites

Despite the previous observations that rutin formed only a limited set of metabolites in fermentations without fibres, we investigated its effect on the whole profile of phenolic acids using a three-way MANOVA with fibre, concentration and rutin addition as factors. The between-subjects effects indicated a significant effect (*p* < 0.05) on six phenolic metabolites: mandelic acid, F(12,1174) = 2.7, *p* = 0.001, vanillic+iso-vanillic acid, F(12,1174) = 3.258, *p* = 0.001; 3,4diOHPAA, F(12,1174) = 2.22, *p* = 0.009; 3,3,4diOHPPA F(12,1174) = 2.01, *p* = 0.020 and *p*-coumaric acid, F(12,1174) = 4.529, *p* = 0.001. The significant interaction for 3,4diOHPAA was investigated by simpler interaction analysis using independent samples t-test (2-tailed) between no fibre added and each fibre concentrations, separately for each timepoint.

**Figure 3:**
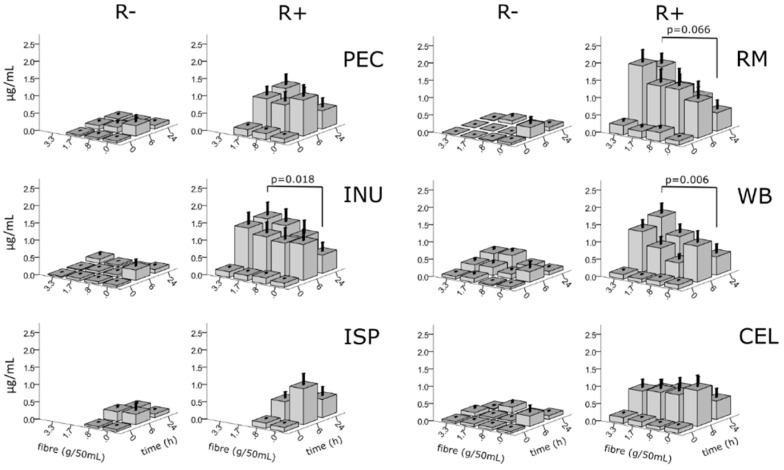
Concentration of 3,4diOHPAA in fermentations in the presence of fibres at four concentrations (0, 0.8, 1.7 and 3.3 g/50 mL) in fermentations with rutin (R+; 20 µg/mL) and without rutin (R-); INU, inulin, PEC, pectin, ISP, ispaghula, RM, pyrodextrin, WB, wheat bran, CEL, cellulose; means ± S.E.M; independent samples t-test (2-tailed), *n* = 10.

WB significantly increased the concentration of 3,4diOHPAA at 24 h, *p* = 0.006 compared to no fibre, with an 2.3-fold increase between blank and the highest concentration tested and INU presence at 24 h facilitated a 2.5-fold increase between blank and the highest concentration *p* = 0.018 and a 2.3-fold increase for the concentration 1.7 g/50 mL (*p* = 0.056) (Figure 3).

**Figure 4:**
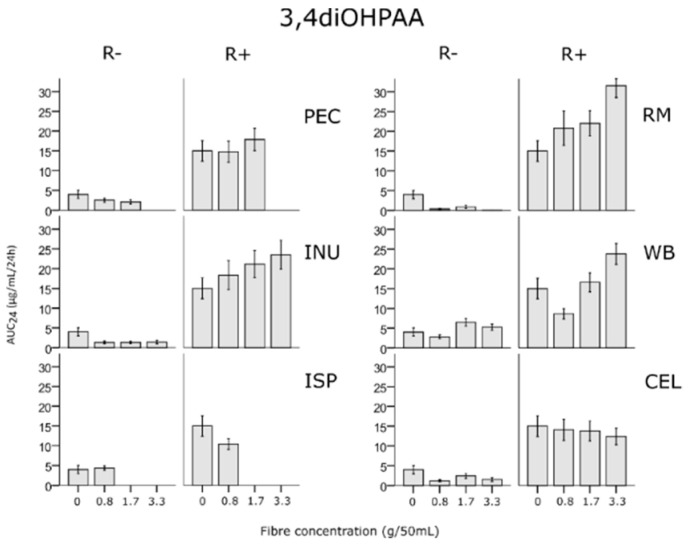
Area under the curve over 24 h of main metabolite 3,4diOHPAA in fermentations with rutin added (R+, 20 µg/mL) and without rutin (R-) as influenced by presence of fibre at four concentrations (0, 0.8, 1.7 and 3.3 g/50 mL); INU, inulin, PEC, pectin, ISP, psyllium, RM, pyrodextrin, WB, wheat bran, CEL, cellulose; means ± S.E.M.

Additionally, there was a 1.6-fold increase for RM at the highest concentration (*p* = 0.066). Plotting the data of individual donors highlighted non-producers of the metabolite (4 of 10), producers (6 of 10 subjects, of which donors 1, 2, 3, 4 and 5 or 50% responded to the addition of fibre with an increased production of 3,4diOHPAA) (Supplementary Materials, Figure S1). The same t-test applied to AUC_24_ data was not significant for any fibre, however the trend for AUC_24_ was similar (Figure 4).

### 3.6. Impact of Fibres and Rutin on pH and SCFA Production

The addition of rutin had a small influence on pH and SCFA profiles and this effect was fibre-specific. In order to provide a generalised and clear message of the influence of fibre in fermentations with the presence of absence of rutin, we carried out an independent samples t-test on AUC_24_ values for SCFA across all fibres and concentrations. The presence of rutin clearly increased propionic acid by 19% (*p* < 0.001), decreased isobutyric acid from 92.4 mmol/L/24 h to 3.8 mmol/L/24 h (*p* < 0.001), increased butyric acid by 12% (*p* = 0.032), valeric acid by 21% (*p* < 0.001) and caproic acid by 44% (*p* = 0.005), for full results, see Table 2.

**Figure 5:**
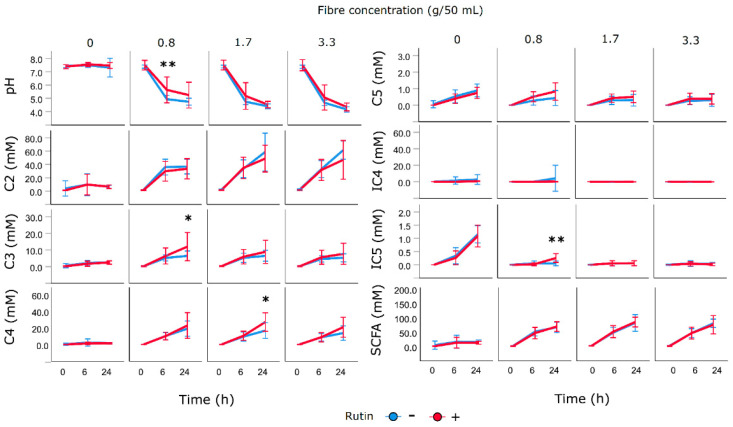
Effect of rutin on formation short chain fatty acid from inulin (3 concentrations) in faecal incubations; * significantly different (*p* < 0.05), ** (*p* < 0.01) between fermentation with rutin (red) and without rutin (blue) using independent samples t-test, adjusted for multiple comparisons, means ± SD for *n* = 10; C2, acetic acid, C3, propionic acid, C4, butyric acid, IC4, isobutyric acid, C5, valeric acid, IC5, isovaleric acid.

The effect of rutin presence on SCFA fermentation profiles can be demonstrated in the case of INU (Figure 5). Rutin presence increased pH at timepoint 6 h (for 0.8 g/50 mL the pH changed from pH 4.94 to pH 5.63 (*p* = 0.010) and a similar, though not significant effect, was seen for other fibre concentrations.

There was no effect on acetic acid, but propionic acid was increased significantly at 24 h for the lowest fibre concentration from 6.47 to 12.01 mmol/L (*p* = 0.032), again, with a similar though not significant effect for both higher fibre concentrations. Rutin also caused an increase of butyric acid at 24 h, from 16.86 to 27.20 mmol/L, (*p* = 0.020) and isovaleric acid at 24 h from 0.056 to 0.251 mmol/L, (*p* = 0.036). In contrast, in the presence of ISP, addition of rutin caused a decrease of acetic acid at 24 h from 23.99 to 36.20 mmol/L (*p* = 0.034), increase of propionic acid from 16.26 to 11.63 mmol/L (*p* = 0.044) and decrease of valeric acid at 24 h from 1.11 to 0.78 mmol/L (*p* = 0.026). Taken together, the data show that rutin slows down the fermentation of fermentable fibres and shifts the SCFA pattern towards production of propionic acid and branched short chain fatty acids. Full data on the effect of rutin on SCFA production of all fibres can be found in Supplementary Materials, Figure S2.

## 4. Discussion

This study aimed to determine if the catabolism of rutin to phenolic acids by the gut microbiota is affected by dietary fibre, We have shown that the type of dietary fibre alters the pattern of rutin catabolism in an in vitro gut fermentation model. Inulin, WB and RM significantly increased the concentrations of the main rutin metabolite, 3,4diOHPAA at 24 h. This may have important consequences on the bioavailability of 3,4diOHPAA and its biological activity. 3,4diOHPAA was previously shown to have a broad spectrum of potentially beneficial activities for mucosal health [15,26,43] and cardiometabolic outcomes [27]. The effect of fibre on phenolic acid production was confirmed using ^13^C isotopically labelled quercetin. Three possible mechanisms of action for this interaction between the fibre and rutin catabolism are proposed: (i) sequestration by some fibres, (ii) changes in microbial communities driven by either substrate availability or antimicrobial action of released phenolics and (iii) saturation/competitive inhibition of microbial enzymes by fibres or their associated phenolic metabolites.

Spiking with a standard compound mixture revealed selective sequestration of (poly)phenolics by some fibres. Quercetin recovery was significantly reduced in the presence of ISP and WB. The phenolic acids were selectively complexed by WB, those with two –OH substitutions of the benzene ring showed a higher affinity than those with one. Methylation reduced the affinity to WB (as seen for ferulic and caffeic acid) while reduction of the side chain increased the affinity (3,3,4diOHPPA vs. caffeic acid). Polyphenol binding capacity thus depends on their molecular weight, conformational mobility and flexibility, water solubility, and joint effects of these three factors [44]. Branched complex polysaccharides offer additional binding sites and together with the degree of the polyphenol B-ring substitution can play a major role in the extent of binding interactions. The high sequestration capacity of WB has been demonstrated for other organic compounds such as bile acids [45] and carcinogens [40]. Complex polysaccharides such as WB [46] and those that form viscous solutions (e.g., PEC) offer additional hydrophobic pockets sequestrating proanthocyanidins in comparison to filamentous or globular polysaccharides (xyloglucan or cellulose) [47].

It is well established that different fibres can selectively promote the populations and activities of individual bacterial species. and previous in vitro studies have suggested that the presence of glucose increases phenolic acid production from quercetin in fermentations [15,19], which is likely to be via its effect on the microbial catabolism or composition. We did not measure bacterial populations in this study but in studies by others, 16S rDNA profiling has previously that the static batch incubation model is useful to mimic colon microbial processes and is widely used to assess the impact of dietary factors on bacterial metabolism in a reproducible fashion for high throughput studies [48]. The abundance of lactobacilli increased in the presence of xylo-oligosaccharides and INU when compared to control without fibre or to the microcrystalline CEL, while resistant starch increased the Bacteroidaecae family. Moreover, in that study, these fibres decreased the relative abundance of the Lachnospiraceae family [48]. Inulin has a demonstrated effect on the growth of bifidobacteria both in vitro [49] and in vivo [50,51,52], highlighting the relevance of batch incubations for initial screening purposes. In another in vitro study, dietary fibres caused specific changes: resistant starch caused a 6-13-fold increase in abundance of *Ruminococcus bromii* and increased the abundance of several species of bifidobacteria. PEC increased bifidobacteria and *F. prausnitzii,* while INU increased *Anaerostipes hadrus* and *Coprococcus. eutactus*; arabinoxylans, which presumably represent the highest fibre fraction of our WB sample increased the abundance of bifidobacteria, mainly of *Bifidobacterum bifidum* and *B. longum* up to 13-times [53].

Changes in microbial profile may play crucial role in phenolic catabolic profile. Microbial strains capable of complete catabolic breakdown of rutin to phloroglucinol, carbon dioxide, 3,4-dihydroxy-benzaldehyde and 3,4diOHPAA include *Butyrivibrio* sp., lactobacilli and some other strains [54]. *Clostridium perfringens*, *Bacteroides fragilis* [55] *Eubacterium ramulus* and *Flavonifractor plautii* showed the ability to produce 3,4diOHPAA from quercetin [55]. Lactobacilli such as *Lactobacillus brevis*, *L*. *fermentum* and *L*. *plantarum* metabolize phenolic acids by decarboxylation and/or reduction, yielding dihydroderivatives or corresponding aldehydes [56]. *L. fermentum* was shown to reduce caffeic acid to dihydrocaffeic acid and similarly *p*-coumaric acid is metabolised by other *Lactobacillus* spp. [57,58]. Changes in abundance of these species and possibly many others, triggered by the presence of fibre, is likely to be responsible for the effect of dietary fibres on rutin catabolism seen in our study. The interplay between fibres, polyphenols and microbiota is very complex. Polyphenols are catabolised in the colon by specific microbial species, they n turn shape gut microbiota composition. This phenomenon has been recently reviewed [59,60].

Evidence from animal models has shown that pectin interacts with polyphenols in an as yet unknown way and that administration in combination (apple polyphenol fraction + fibre fraction) was more effective in preventing atherosclerosis in a mouse model than apple polyphenol fraction or fibre fraction only [61] or that plasma levels of quercetin are higher in rats fed a rutin-pectin-diet than on a rutin-cellulose diet or basal diet with rutin [62,63].

Very few studies have investigated the fate of rutin or quercetin at physiological concentrations, [14,30] with other studies considering doses achievable only by supplements [15]. Our results differ from studies using higher doses of the parent compounds in that they report slower deglycosylation, [13] or observe 3,4diOHBA, 3,3OHPPA and 3,3,4diOHPPA and other metabolites alongside the four metabolites observed in the present study [13,15,64]. Studies with high doses also report higher recoveries of total metabolites to initial quercetin and longer lag time in decomposition of the parent compound, which may be due to saturation of microbial hydrolases and slower release of the aglycone or growth inhibition of selected intestinal bacterial species [65].

In our study, almost all initial rutin aglycone was degraded during the first 6 h, whereas in another study, most of the rutin (336 µg/mL) was still intact after 6 h fermentation [15]. This probably reflects the fact that in our study, the rutin dose was much lower, close to physiological intake. The fibres used here were themselves a good source of phenolic acids probably related to the non-extractable polyphenols. Phenolic precursors of these metabolites are an inevitable component of cell wall structures, bridging and cross-linking structural carbohydrate polymers and may differ among plants [46].

Fibre intake in clinical trials is linked to the presence of phenolic metabolites in stool, such as ferulic acid, 3,4OH3MPPA and 3OHPPA [66]. These aromatic compounds can also derive from the phenylalanine, tyrosine and tryptophan degradation [67] forming in particular, PAA, 4OHPAA and indole-3-acetic acid [68]. These catabolic pathways exist in parallel and interact, suggesting a considerable role of the food matrix in polyphenol metabolism. For example, ferulic acid catabolism to 3OHPAA and 3,4OHPAA was reduced in vitro in subjects on a high-fat-high protein diet [69].

Our study showed which aromatic acids originated from protein catabolism, such as those occurring in blank-medium only and those present in vials with cellulose (mainly PAA, 3PPA and less 4OHPAA, 3,3OHPPA and 3,4OHPPA, those associated with the fibre and probably originating from plant-derived phenylpropanoids and amino acids (Figure 2). The presence of specific metabolites such as phenyllactic acid in INU fermentations is of interest and suggesting a specific precursor in the fibre. The 3-Phenyllactic acid is a potent antimicrobial agent used in the cheese industry and is produced by several strains of lactic acid bacteria, such as human colonic *L. rhamnosus*, from phenylalanine [70]. It promotes growth of some bacterial groups [71] and may act in *quorum sensing* [72,73]. The presence of this metabolite could contribute to the prebiotic effect of inulin.

The presence of pectin led to production of 3,3OH4MPPA and 3,3OHPPA while WB was associated with high abundance of PAA and 3,3OHPPA, but also contained ferulic acid and 3,4OH3MPPA (Table 1, Figure 2). This suggests different structural properties or different precursors. Interestingly, *m*-hydroxy dihydro-catabolites of rutin could be more resistant to further catabolism as microbial enzymes show preference towards faster removal of the hydroxyl or *O*-methoxy group in the *para*-position [74], explaining their higher proportion.

The addition of rutin influences carbohydrate fermentation. Fermentation of fibres to SCFA is inhibited by rutin and the branched chain fatty acids increase in response to this, possibly originating from non-carbohydrate sources. Except for RM, rutin slightly increased pH in fermentations of all fibres including CEL, suggesting a slight effect on carbohydrate metabolism or microbiota profile even at the low, physiologically relevant concentrations used. Polyphenols have been shown to have potential to alter gut microecology by a prebiotic-like effect; selectively inhibiting the growth of certain bacterial species [65]. Isobutyric acid, for instance is produced by *Odoribacter* sp. [75] so such marked decrease may denote an effect on this or a similar microbial species.

## 5. Conclusions

In this study, dietary fibres showed differential interactions with dietary rutin which affected the pattern of phenolic acid and SCFA production. Wheat bran, pectin, psyllium, resistant maltodextrin, but not cellulose, were a rich source of phenolic acids producing a profile with some unique metabolites, characteristic for each fibre. Rutin at the physiological dose tested was catabolised to four main phenolic acids. Addition of inulin, an analogue to 20 g bolus dietary intake increased the concentration of the main bioactive metabolite, 3,4diOHPAA in fermentations at 24 h 2.3-fold and addition of wheat bran 2.5-fold. Moreover, the area under the curve of this metabolite tended to be higher in fermentations with fibre, suggesting higher bioaccessibility. Understanding these interactions is essential as fibre and polyphenols are often eaten together and the phenolic acids and SCFA produced may be important effectors for key health benefits. In vitro fermentation models have some limitations in closely modelling events in the human colon as they do not mimic absorption of bacterial products but they are useful for predicting microbial activity. The interactions between fibre, polyphenols and the gut microbiota need to be further studied in vivo.

## Figures and Tables

**Table 1:** Area under the curve (AUC_24_) of phenolics found in fermentation slurries with fibres only (rutin not added) at 0.8 g/50 mL, AUC_24_ µg/mL/24 h.

	Fibre						
	PEC	INU	ISP	RM ^#^	WB	CEL	Blank ^##^
	Mean ± SEM	Mean ± SEM	Mean ± SEM	Mean ± SEM	Mean ± SEM	Mean ± SEM	Mean ± SEM
BA	37.1 ± 4.4	49.7 ± 6.6	**37.7** **±** **5.3** ^*^	78.4 ± 15.7	48.0 ± 4.6	35.1 ± 6.1	33.3 ± 3.7
PAA	245.6 ± 20.9 ^**^	205.1 ± 15.4 ^**^	267.3 ± 19.1 ^**^	233.2 ± 16.7 ^**^	649.4 ± 53.6	510.7 ± 37.8	598.5 ± 40.9
Pyrocatechol	20.6 ± 4.5	**58.7** **±** **8.6** ^*^	16.8 ± 3.2	**43.3** **±** **2.9** ^**^	**38.3** **±** **5.4** ^**^	23.4 ± 5.2	15.3 ± 3.8
Resorcinol	8.0 ± 2.7	6.2 ± 2.5	7.2 ± 2.6	0.0 ± 0.0	9.2 ± 2.6	6.4 ± 2.2	5.1± 1.8
3PPA	191.6 ± 21.6	139.6 ± 16.5	136.3 ± 11.8	129.3 ± 5.0	280.6 ± 32.8	139.6 ± 16.6	118.3 ± 12.4
Mandelic acid	5.3 ± 1.4	4.4 ± 1.2	**10.3** **±** **1.8** ^**^	1.4 ± 0.6	**10.4** **±** **1.8** ^*^	3.1 ± 0.8	2.5 ± 0.6
Cinnamic acid	**4.6** **±** **1.7** ^*^	2.8 ± 1.4	2.2 ± 1.3	0.0 ± 0.0	2.3 ± 1.2	1.8 ± 1.0	1.9 ± 1.0
3HBA	3.2 ± 1.5 ^*^	6.6 ± 3.3	6.3 ± 3.0	10.7 ± 2.3	5.9 ± 3.0	3.7 ± 1.6	3.9 ± 2.0
3-Phenyllactic acid	34.5 ± 6.6	**120.6** **±** **15.5** ^**^	11.9 ± 5.1	22.1 ± 9.9	7.2 ± 1.7	2.4 ± 0.6	4.6 ± 1.5
3OHPAA	7.0 ± 1.2	7.1 ± 1.3	10.7 ± 1.4	8.9 ± 2.4	7.6 ± 1.2	6.7 ± 1.2	4.5 ± 0.8
4OHBA	3.7 ± 0.6	4.9 ± 1.0	6.8 ± 0.8	**10.4** **±** **1.8** ^*^	**12.8** **±** **1.1** ^**^	6.1 ± 1.2	2.8 ± 0.6
Phloroglucinol	3.8 ± 1.3	10.9 ± 3.1	7.3 ± 2.0	6.4 ± 2.8	7.1 ± 2.1	6.4 ± 2.1	7.9 ± 1.8
4OHPAA	14.1 ± 1.4 ^**^	16.8 ± 2.3 ^**^	12.4 ± 1.5 ^**^	14.3 ± 3.1 ^**^	62.4 ± 4.1	52.2 ± 3.7	50.0 ± 4.0
3,3OHPPA	**115.3** **±** **8.5** ^**^	26.2 ± 3.9 ^**^	37.3 ± 4.7	11.6 ± 3.4 ^**^	**128.5** **±** **17.7** ^**^	36.3 ± 4.3	40.9 ± 6.8
VAN + iso-VAN	0.9 ± 0.3	1.2 ± 0.3	3.1 ± 0.6	2.4 ± 1.0	**7.3** **±** **0.7** ^*^	1.2 ± 0.3	0.8 ± 0.2
4-OHmandelic acid	2.6 ± 1.2	2.9 ± 1.1	1.6 ± 0.7	0.0 ± 0.0	1.8 ± 0.7	3.2 ± 1.0	0.6 ± 0.1
3OH4MPAA + 4OH3MPAA	7.2 ± 2.3	5.2 ± 1.5	7.1 ± 2.6	15.5 ± 6.9	11.1 ± 2.9	11.6 ± 4.3	6.1 ± 2.6
3,4OHPPA	23.2 ± 3.4	16.9 ± 3.5	18.5 ± 3.6	14.1 ± 4.8	26.0 ± 3.7	32.3 ± 5.9	27.6 ± 5.5
3,4diOHBA	1.0 ± 0.2	2.3 ± 0.4	1.5 ± 0.2	**3.3** **±** **0.5** ^**^	**5.3** **±** **0.5** ^**^	1.0 ± 0.3	3.1 ± 1.5
3,4diOHPAA	2.5 ± 0.5	1.3 ± 0.4	**4.3** **±** **0.6** ^**^	0.4 ± 0.2	2.8 ± 0.5	1.2 ± 0.3	4.0 ± 1.0
3,3OH4MPPA	**132.5** **±** **12.0** ^**^	0.5 ± 0.1 ^**^	0.6 ± 0.2	0.0 ± 0.0	0.4 ± 0.2	0.4 ± 0.2	5.0 ± 2.1
3,4OH3MPPA	2.1 ± 0.6	1.5 ± 0.6	1.8 ± 0.9 ^**^	0.0 ± 0.0 ^**^	**26.3** **±** **3.2** ^**^	2.1 ± 0.6	1.0 ± 0.4
3,3,4diOHPPA	12.2 ± 3.7	4.0 ± 2.0	8.9 ± 2.6	2.2 ± 1.0	**9.6** **±** **1.6** ^**^	6.2 ± 2.3	5.8 ± 1.8
*p*-Coumaric acid	0.2 ± 0.1	0.3 ± 0.1	0.4 ± 0.1	0.0 ± 0.0	0.5 ± 0.1	0.1 ± 0.0	0.4 ± 0.1
3OH4M Cinnamic acid	0.5 ± 0.3	0.1 ± 0.1	0.4 ± 0.1	0.0 ± 0.0	0.5 ± 0.2	0.0 ± 0.0	0.1 ± 0.1
Ferulic acid	0.2 ± 0.1	0.2 ± 0.1	0.6 ± 0.2	0.0 ± 0.0	**5.2** **±** **0.7** ^**^	0.4 ± 0.1	0.6 ± 0.2
Caffeic acid	4.6 ± 2.0	4.2 ± 2.1	5.7 ± 1.9	0.8 ± 0.4	3.6 ± 1.0	2.9 ± 1.0	2.7 ± 1.3
Quercetin	6.0 ± 3.3	7.0 ± 3.8	**11.7** **±** **2.9** ^**^	0.0 ± 0.0	7.8 ± 3.2	3.0 ± 1.6	4.4 ± 1.8

* significantly different at *p* < 0.05 from CEL; ** significantly different at *p* < 0.01 from CEL using multivariate ANOVA with Dunnett’s post hoc test; *n* = 10 except (^#^, *n* = 2, ^##^, *n* = 17); S.E.M, standard error of mean; values in **bold** refer to a significant increase of the analyte in comparison to the blank; INU, inulin, PEC, pectin, ISP, psyllium, RM, pyrodextrin, WB, wheat bran, CEL, cellulose.

**Table 2:** Effect of rutin on short chain fatty acid profile expressed as AUC_24_ irrespective of concentrations and type of fibre (ISP at concentration 0.8 g/50 mL, PEC at 0.8 and 1.7 g/50 mL, CEL, INU, RM at 0.8, 1.7 and 3.3 g/50 mL).

AUC_24_	Rutin not Added	Rutin Added	*p*-Value	*n* (Total)
	Mean ± S.D.	Mean ± S.D.		
pH	147.11 ± 19.42	148.68 ± 19.59	0.187	300
C2	617.79 ± 351.04	618.37 ± 348.00	0.980	300
**C3**	**120.48 ± 66.61**	**143.05 ± 88.11**	**0.000**	**300**
**IC4**	**92.24 ± 344.67**	**3.83 ± 3.83**	**0.000**	**300**
**C4**	**202.45 ± 167.81**	**226.58 ± 177.94**	**0.032**	**300**
IC5	6.80 ± 6.57	6.87 ± 6.45	0.874	300
**C5**	**11.80 ± 8.15**	**14.35 ± 8.22**	**0.000**	**300**
IC6	0.01 ± 0.13	0.01 ± 0.11	0.574	300
**C6**	**2.50 ± 5.21**	**3.61 ± 6.64**	**0.005**	**300**
C7	0.12 ± 0.62	0.16 ± 0.67	0.361	300
Sum C2-C5	1051.56 ± 578.89	1013.04 ± 516.17	0.285	300

Independent samples t-test (2-tailed) was used. For abbreviations and AUC_24_ explained, see Materials and methods section. Values except pH are expressed in mmol/L/24 h. Statistically significant values (*p* < 0.05) are in **bold**.

## Supplementary Materials

The following are available online at https://www.mdpi.com/2072-6643/12/6/1577/s1, Table S1: Overview of the chemicals used in the study with their abbreviations Table S2: Basic method validation and GC-MS of the TMS derivatives of phenolic metabolites in batch incubations after spiking the sample with two concentrations of standards, Table S3: Recoveries of metabolites (µg/mL) spiked with 5 µg/mL of standard into aqueous suspensions of fibres (concentration equals that used in test) with an aim to investigate fibre sequestration properties, Figure S1: Inter-individual differences in the production of 3,4diOHPAA from rutin among donors and response to the presence of fibres, Figure S2: Effect of rutin on formation of short chain fatty acid from fibres (3 concentrations) in faecal incubations, Figure S3: Information sheet on the low polyphenol diet provided the stool donors.
