# Supplementary material for: Dietary Fibres Differentially Impact on the Production of Phenolic Acids from Rutin in an In Vitro Fermentation Model of the Human Gut Microbiota
Source: Nutrients. 2020 May 28;12(6):1577. doi: 10.3390/nu12061577 (PMC7352394; doi:10.3390/nu12061577)
Supplement: Supplementary file 1 [file nutrients-12-01577-s001.docx]

**Supporting information**

Havlik J., Marinello V., Gardyne A., Hou M., Mullen W., Morrison D.J., Preston T., Combet E., Edwards C.A. Dietary fibres differentially impact on the production of phenolic acids from rutin in an *in vitro* fermentation model of the human gut microbiota

**Supporting information Table  S1** Overview of the chemicals used in the study with their abbreviations 

| Chemical name | Abbreviation used | Manufacturer |
| --- | --- | --- |
| 2,4,5-trimethoxycinnamic acid | TMCA | Sigma-Aldrich |
| 3-(3-hydroxy-4-methoxyphenyl)propionic acid | 3,3OH4MPPA | Sigma-Aldrich |
| 3-(3-hydroxyphenyl)propionic acid | 3,3OHPPA | Alfa Aesar |
| 3-(4-hydroxy-3-methoxyphenyl)propionic acid | 3,4OH3MPPA | Sigma-Aldrich |
| 3-(4-hydroxyphenyl)propionic acid | 3,4OHPPA | Sigma-Aldrich |
| 3-3,4-dihydroxyphenyl)propionic acid | 3,3,4diOHPPA | Alfa Aesar |
| 3-hydroxy-4-methoxycinnamic acid | Isoferulic acid | Sigma-Aldrich |
| 3-hydroxy-4-methoxyphenylacetic acid | 3OH4MPAA | Sigma-Aldrich |
| 3-hydroxybenzoic acid | 3OHBA | Sigma-Aldrich |
| 3-hydroxyphenylacetic acid | 3OHPAA | Sigma-Aldrich |
| 3-phenyllactic | 3PLA | Sigma-Aldrich |
| 3-phenylpropionic acid | 3PPA | Sigma-Aldrich |
| 3,4-dihydroxybenzoic acid | 3,4OHBA | Sigma-Aldrich |
| 3,4-dihydroxyphenylacetic acid | 3,4diOHPAA | Sigma-Aldrich |
| 4-hydroxy-3-methoxybenzoic acid | VAN | Sigma-Aldrich |
| 4-hydroxy-3-methoxyphenylacetic acid | 4OH3MPAA | Alfa Aesar |
| 4-hydroxybenzoic acid | 4OHBA | Sigma-Aldrich |
| 4-hydroxymandelic acid | 4OH-mandelic acid | Alfa Aesar |
| 4-hydroxyphenyl acetic acid | 4OHPAA | Sigma-Aldrich |
| benzoic acid | BA | Sigma-Aldrich |
| caffeic acid | - | Sigma-Aldrich |
| ferulic acid | - | Sigma-Aldrich |
| mandelic acid, | - | Sigma-Aldrich |
| *p*-coumaric acid | - | Sigma-Aldrich |
| phenylacetic acid | PAA | Sigma-Aldrich |
| phloroglucinol | - | Sigma-Aldrich |
| pyrocatechol | - | Sigma-Aldrich |
| quercetin | - | Sigma-Aldrich |
| resorcinol | - | Sigma-Aldrich |
| *trans*-cinnamic acid | - | Sigma-Aldrich |

**Supporting information Table S2** Basic method validation and GC-MS of the TMS derivatives of phenolic metabolites in batch incubations after spiking the sample with two concentrations of standards

| Metabolite | RT | Quantifier ions | Recovery | | LOD |
| --- | --- | --- | --- | --- | --- |
|  |  | (m/z^+^) | 10 µg/mL | 2 µg/mL | (µg/mL) |
| BA | 2.34 | 105 | 124 | 148 | 1.5 |
| PAA | 2.65 | 164 | 54 | ND | 0.3 |
| Pyrocatechol | 2.81 | 254 | 100 | 55 | 1.0 |
| Resorcinol | 3.31 | 239; 254 | 26 | - | 0.5 |
| 3PPA | 3.71 | 104; 222 | 119 | 42 | 0.3 |
| Mandelic acid | 4.21 | 147; 179 | 86 | 72 | 1.5 |
| Cinnamic acid | 5.20 | 103; 161; 205 | 90 | 62 | 0.35 |
| 3OHBA | 5.36 | 193; 223; 267 | 91 | 69 | 0.8 |
| 3-Phenyllactic | 5.59 | 193 | 90 | 51 | 1.5 |
| 3OHPAA | 5.95 | 164 | 91 | 68 | 0.2 |
| 4OHBA | 6.25 | 267; 282 | 90 | 65 | 0.25 |
| Phloroglucinol | 6.45 | 327; 242 | 10 | - | 1.9 |
| 4OHPAA | 6.42 | 164,252 | 90 | 60 | 0.3 |
| 3,3OHPPA | 7.82 | 177; 192; 205; 310 | 98 | 79 | 0.15 |
| 3,4OHPPA | 8.36 | 192; 310 | 93 | 77 | 0.35 |
| VAN+ iso-VAN | 8.40 | 223; 297 | 87 | 68 | 0.4 |
| 4-Hydroxymandelic acid | 8.66 | 341; 267 | 61 | 92 | 2.5 |
| 4OH3MPAA+3OH4MPAA | 8.50 | 311; 326 | 93 | 77 | 0.4 |
| 3,4diOHBA | 9.35 | 193; 211; 355; 370 | 92 | 83 | 0.03 |
| 3,4diOHPAA | 9.48 | 267; 384 | 95 | 83 | 0.08 |
| 3,3OH4MPPA | 10.48 | 192; 209 | 95 | 77 | 0.3 |
| 3,4OH3MPPA | 10.65 | 192; 209; 310; 340 | 81 | 42 | 0.4 |
| 3,3,4diOHPPA | 11.53 | 179; 267; 398 | 83 | 43 | 0.25 |
| *p*-Coumaric acid | 11.46 | 219; 249; 293; 308 | *84* | 60 | 0.5 |
| 3OH4M Cinnamic acid | 13.80 | 308; 338 | 94 | 82 | 0.5 |
| Ferulic acid | 14.00 | 249; 293; 308; 323 338 | 106 | 118 | 1.1 |
| Caffeic acid | 14.70 | 219; 396 | 101 | 97 | 2 |
| TMCA | 15.65 | 89 | 94 | 96 | ND |
| Quercetin | 23.17 | SIM 647-649 | 83 | 43 | 1.0 |

ND, not determined. For abbreviations, see section Materials and Methods

**Supporting information Table S3** Recoveries of metabolites (µg mL^-1^) spiked at 5 µg mL^-1^ into aqueous suspensions of fibres (concentration equals that used in test) with an aim to investigate fibre sequestration properties

|  | Fibre | | | | | | | | | | | | | | | |
| --- | --- | --- | --- | --- | --- | --- | --- | --- | --- | --- | --- | --- | --- | --- | --- | --- |
|  | PEC | | INU | | | ISP | RM | | | WB | | | CEL | | | BLANK |
|  | Fibre doncentration (g / 50 mL) | | | | | | | | | | | | | | | |
|  | 0.8 | 1.7 | 0.8 | 1.7 | 3.3 | 0.8 | 0.8 | 1.7 | 3.3 | 0.8 | 1.7 | 3.3 | 0.8 | 1.7 | 3.3 | 0 |
| PAA | 9.8 | 10.5 | 6.0 | 7.4 | 7.1 | 7.4 | 6.6 | 5.9 | 5.9 | 6.9 | 9.0 | 8.9 | 5.1 | 5.8 | 7.7 | 9.7 |
| 3PPA | 7.0 | 9.0 | 5.7 | 6.4 | 5.9 | 5.8 | 5.7 | 4.8 | 5.5 | 5.6 | 6.4 | 8.0 | 5.4 | 5.7 | 6.4 | 7.6 |
| Cinnamic acid | 7.9^*^ | 8.7^*^ | 6.4 | 6.0 | 6.3 | 6.5 | 5.7 | 5.3 | 5.5 | 5.6 | 6.1 | 6.2 | 5.0 | 5.4 | 6.6 | 6.8 |
| 3OHBA | 9.5^*^ | 9.1^*^ | 5.8 | 5.7 | 6.1 | 6.1 | 5.8 | 4.9 | 5.5 | 5.5 | 5.2 | 5.2 | 4.7 | 5.2 | 6.2 | 5.3 |
| 3-Phenylllactic | 10.6^*^ | 11.7^*^ | 5.9 | 5.8 | 5.0 | 6.1 | 5.1 | 5.2 | 5.8 | 4.7 | 5.5 | 4.8 | 4.2 | 5.5 | 6.5 | 5.6 |
| 3OHPAA | 7.5^*^ | 8.9^*^ | 5.7 | 5.4 | 5.6 | 5.2 | 6.0 | 4.5 | 5.8 | 5.1 | 5.1 | 5.0 | 5.0 | 5.5 | 6.1 | 5.6 |
| 4OHBA | NF | 8.9^*^ | 5.9 | 5.2 | 6.4 | 6.2 | 5.5 | 5.1 | 5.4 | 5.9 | 5.8 | 5.8 | 4.8 | 5.1 | 6.2 | 5.5 |
| 4OHPAA | 7.9^*^ | 8.6^*^ | 6.1 | 5.8 | 5.9 | 6.8 | 5.8 | 5.4 | 6.0 | 4.4 | 4.1^*^ | 2.0^*^ | 4.7 | 5.4 | 6.1 | 5.7 |
| 3,3OHPPA | 8.0^*^ | 8.7^*^ | 6.0 | 5.6 | 5.7 | 6.3 | 5.7 | 5.4 | 5.7 | 5.0 | 5.0 | 5.2 | 4.9 | 5.3 | 5.7 | 5.8 |
| Mandelic_acid | 9.3^*^ | 9.1^*^ | 6.0 | 5.9 | 6.1 | 6.6 | 5.7 | 6.3 | 6.1 | 3.5^*^ | 2.2^*^ | 0.8^*^ | 4.8 | 5.6 | 5.5 | 5.5 |
| 3OHPPA | 8.8^*^ | 8.8^*^ | 5.9 | 5.8 | 5.8 | 6.5 | 5.9 | 6.2 | 5.9 | 3.3^*^ | 2.1^*^ | 0.7^*^ | 4.9 | 5.4 | 5.7 | 5.3 |
| VAN_+_*iso*-VAN | 7.4^*^ | 7.7^*^ | 4.9 | 5.6 | 6.2 | 5.5 | 5.5 | 5.5 | 4.6 | 6.1 | 4.9 | 5.0 | 5.0 | 5.1 | 5.2 | 5.6 |
| 4-hydroxy_mandelic_acid | 4.3 | 7.6 | 6.3 | 7.0 | 5.0 | 6.0 | 4.4 | 5.3 | 7.2 | 5.2 | 5.1 | 5.1 | 5.2 | 6.1 | 8.1^*^ | 5.6 |
| 3OH4MPAA_+_4OH3MPAA | 8.7^*^ | 9.7^*^ | 5.4 | 5.1 | 6.2 | 5.5 | 5.4 | 5.0 | 5.1 | 4.5 | 4.7 | 4.2 | 4.2 | 4.6 | 5.2 | 4.7 |
| 3,4diOHBA | NF | NF | 5.6 | 5.7 | 5.9 | 6.2 | 5.2 | 5.0 | 5.3 | 5.4 | 5.2 | 4.5 | 5.1 | 5.0 | 6.5 | 5.3 |
| 3,4diOHPAA | 8.5^*^ | 8.4^*^ | NF | 5.8 | 6.3 | 6.1 | 5.2 | 5.3 | 5.5 | 0.4^*^ | 0.7^*^ | 0.6^*^ | 4.8 | 5.3 | 6.0 | 5.8 |
| 3,3OH4MPPA | 7.9^*^ | 7.8^*^ | 5.5 | 5.3 | 5.9 | 5.8 | 5.6 | 5.3 | 5.1 | 5.2 | 4.8 | 4.7 | 4.8 | 5.2 | 5.2 | 4.5 |
| 3,4OH3MPPA | 8.6^*^ | 8.9^*^ | 5.1 | 6.0 | 5.8 | 5.7 | 5.0 | 5.1 | 5.5 | 4.6 | 5.4 | 5.1 | 4.6 | 5.4 | 5.7 | 5.1 |
| 3,3,4diOHPPA | 8.1^*^ | 7.9^*^ | 5.3 | 5.7 | 6.3 | 5.7 | 5.1 | 5.1 | 5.2 | 1.6^*^ | 0.8^*^ | 0.1^*^ | 4.8 | 5.1 | 5.7 | 5.2 |
| p-coumaric_acid | 7.6^*^ | 8.8^*^ | 5.6 | 5.8 | 5.9 | 5.9 | 5.7 | 5.9 | 5.9 | 2.5^*^ | 1.0^*^ | 0.2^*^ | 5.1 | 5.3 | 6.0 | 5.6 |
| 3OH4M_cinnamic_acid | 8.1^*^ | 10.7^*^ | 5.3 | 5.3 | 5.9 | 7.3 | 4.3 | 4.9 | 5.4 | 5.4 | 6.5 | 5.3 | 4.7 | 5.1 | 5.7 | 5.7 |
| Ferulic_acid | 8.4^*^ | 8.3^*^ | 5.6 | 5.8 | 5.6 | 6.8 | 5.2 | 5.1 | 5.3 | 4.9 | 4.9 | 6.3 | 5.4 | 5.1 | 6.3 | 4.7 |
| Caffeic_acid | 7.8^*^ | 9.0^*^ | 5.4 | 5.3 | 5.4 | 5.8 | 5.3 | 5.1 | 5.3 | 3.9^*^ | 2.8^*^ | 1.0^*^ | 5.5 | 4.6 | 5.6 | 5.2 |
| Quercetin | 5.6 | 5.0 | 4.7 | 5.4 | 6.3 | 4.0 | 5.1 | 5.2 | 5.5 | 0.7^*^ | 0.1^*^ | 0.1^*^ | 5.1 | 5.0 | 5.8 | 5.2 |

Each number represent a mean of three replicates, INU, inulin, PEC, pectin, ISP, ispaghula, RM, pyrodextrin, WB, wheat bran, CEL, cellulose; NF, not found


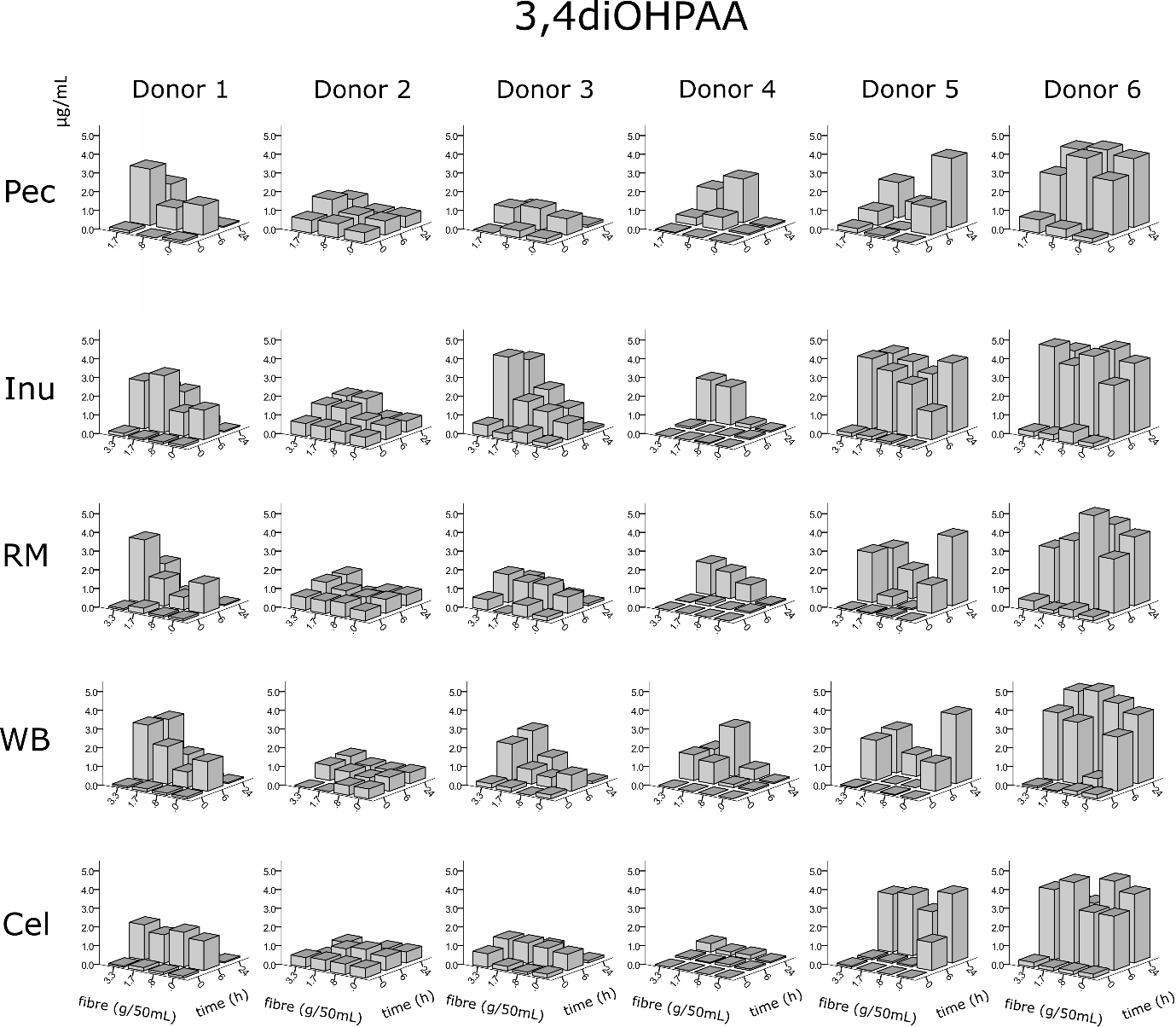


**Supporting information Figure S1** Inter-individual differences in the production of 3,4diOHPAA from rutin among donors and response to the presence of fibres. Each bar represents a separate biological replicate. Donor 3 was used in the ^13^C experiment.

Inu, inulin, Pec, pectin, Isp, ispaghula, RM, pyrodextrin, WB, wheat bran, Cel, cellulose

|  |  |
| --- | --- |
|  |  |

|  |  |
| --- | --- |
|  |  |

**Supporting information Figure S2** Effect of rutin on formation short chain fatty acid from fibres (3 concentrations) in faecal incubations

^*^, significantly different (*p <* 0.05), ^**^ (*p <* 0.01) between fermentation with- (red) and without rutin (blue) using Students t-test, adjusted for multiple comparisons, means ± S, *n* = 10, INU, inulin, PEC, pectin, ISP, ispaghula, RM, pyrodextrin, WB, wheat bran, CEL, cellulose, C2, acetic acid, C3, propionic acid, C4, butyric acid, IC4, isobutyric acid, C5, valeric acid, IC5, isovaleric acid, C6, caproic acid. Rutin +/-, rutin (20 µg mL^-1^) added/without rutin.

**Supporting information Figure S3** Information sheet on the low polyphenol diet provided the stool donors. Compliance was verified with an additional 24 h dietary assessment.
